# Advancing genome editing with artificial intelligence: opportunities, challenges, and future directions

**DOI:** 10.3389/fbioe.2023.1335901

**Published:** 2024-01-08

**Authors:** Shriniket Dixit, Anant Kumar, Kathiravan Srinivasan, P. M. Durai Raj Vincent, Nadesh Ramu Krishnan

**Affiliations:** ^1^ School of Computer Science and Engineering, Vellore Institute of Technology, Vellore, India; ^2^ School of Bioscience and Technology, Vellore Institute of Technology, Vellore, India; ^3^ School of Computer Science Engineering and Information Systems, Vellore Institute of Technology, Vellore, India

**Keywords:** genome editing and CRISPR/Cas9, base editing and AI, AI models for gRNA design, prime editing and AI, genome editing outcome prediction using AI, genomics and AI, off-target prediction, precision medicine and AI

## Abstract

Clustered regularly interspaced short palindromic repeat (CRISPR)-based genome editing (GED) technologies have unlocked exciting possibilities for understanding genes and improving medical treatments. On the other hand, Artificial intelligence (AI) helps genome editing achieve more precision, efficiency, and affordability in tackling various diseases, like Sickle cell anemia or Thalassemia. AI models have been in use for designing guide RNAs (gRNAs) for CRISPR-Cas systems. Tools like DeepCRISPR, CRISTA, and DeepHF have the capability to predict optimal guide RNAs (gRNAs) for a specified target sequence. These predictions take into account multiple factors, including genomic context, Cas protein type, desired mutation type, on-target/off-target scores, potential off-target sites, and the potential impacts of genome editing on gene function and cell phenotype. These models aid in optimizing different genome editing technologies, such as base, prime, and epigenome editing, which are advanced techniques to introduce precise and programmable changes to DNA sequences without relying on the homology-directed repair pathway or donor DNA templates. Furthermore, AI, in collaboration with genome editing and precision medicine, enables personalized treatments based on genetic profiles. AI analyzes patients’ genomic data to identify mutations, variations, and biomarkers associated with different diseases like Cancer, Diabetes, Alzheimer’s, etc. However, several challenges persist, including high costs, off-target editing, suitable delivery methods for CRISPR cargoes, improving editing efficiency, and ensuring safety in clinical applications. This review explores AI’s contribution to improving CRISPR-based genome editing technologies and addresses existing challenges. It also discusses potential areas for future research in AI-driven CRISPR-based genome editing technologies. The integration of AI and genome editing opens up new possibilities for genetics, biomedicine, and healthcare, with significant implications for human health.

## 1 Introduction

**FIGURE 1:**
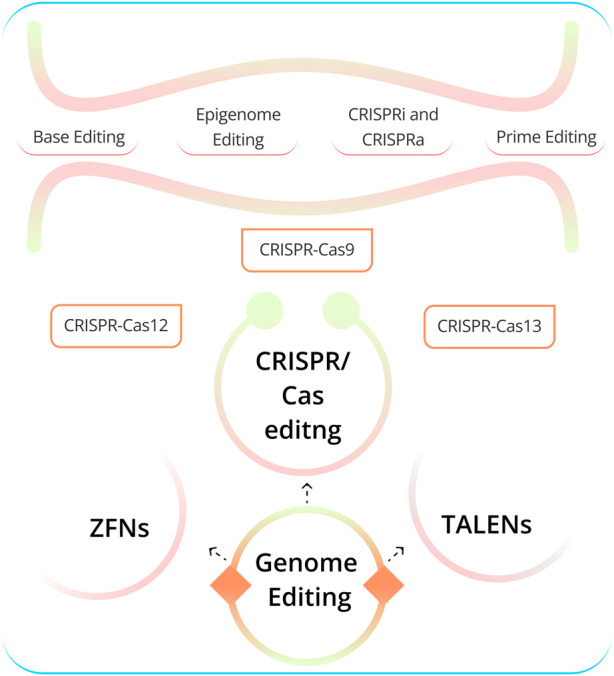
Various aspects of GED. CRISPR encompasses various gene-editing approaches. The most well-known is CRISPR-Cas9, utilizing Cas9 endonuclease guided by sgRNA to target and modify specific DNA sequences. Numerous other Cas nucleases have been identified in the recent past. CRISPR-Cas12 offers a different target site and has diagnostic advantages. Cas13 functions as an RNA-guided RNA endonuclease, specifically targeting and cleaving RNA. Base editing allows precise changes without double-strand breaks, while Prime editing enables versatile DNA sequence modifications. Epigenome editing controls gene expression via epigenetic marks, while CRISPRi and CRISPRa regulate gene expression without altering DNA. These techniques each have distinct applications in genetics and medical research, selected based on specific objectives and genetic contexts. [TALENs: Transcription activator-like effector nucleases, ZFNs: Zinc-finger nucleases, sgRNA: single-guide RNA (Abbreviation: Table 1)].

**FIGURE 2:**
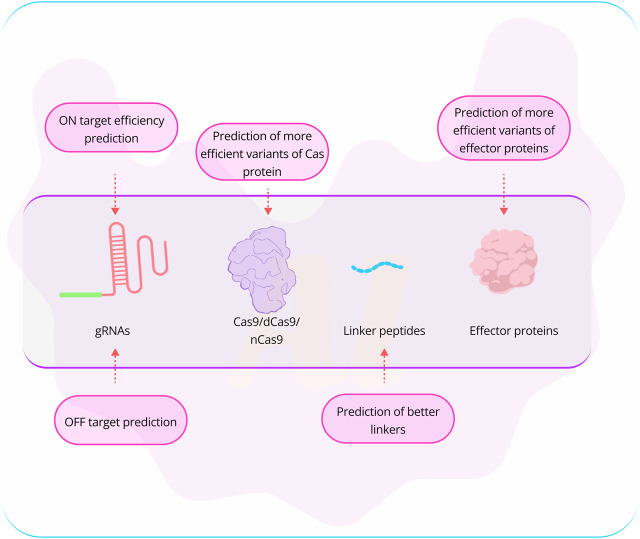
Challenges associated with genome editing using CRISPR technology. One of the most acknowledged challenges in CRISPR-based genome editing is the efficiency of on-target editing and the potential off-target effects. Researchers have explored the use of AI-based tools, such as AlphaFold2, for the prediction of more effective Cas variants and effector proteins. While designing linker peptides is currently not a major obstacle, it remains crucial when connecting an effector protein to a Cas nuclease. (Abbreviation: Table 1).

Genome editing (GED) technologies allow for the precise alteration of DNA sequences in living cells (Ma and Liu, 2015). This has transformed our ability to study gene functionality and develop new therapeutic strategies. The three most advanced GED technologies (Figure 1) are zinc-finger nucleases (ZFNs), transcription activator-like effector nucleases (TALENs), and CRISPR-Cas-associated nucleases (CRISPR/Cas9) (Gaj et al., 2013; Gaj et al., 2016; Siva et al., 2021). CRISPR/Cas9 is the most commonly used GED technology due to its versatility, effectiveness, and ease of use (Zhu, 2022; Adli, 2018; Arora and Narula, 2017). The cell and gene therapy sector are constantly evolving, and recent years have seen remarkable progress in the creation of CRISPR-based treatments, leading to the commencement of numerous clinical trials (CTG Labs - NCBI, 2023a; CTG Labs - NCBI, 2023b; CTG Labs - NCBI, 2023c; CTG Labs - NCBI, 2023d). GED technologies can be used to treat human diseases in a number of ways (Li et al., 2020). For example, it can be employed to address disease-causing mutations, such as those in tumor suppressor genes or cardiovascular diseases like long QT syndrome and hypertrophic cardiomyopathy. Additionally, it can be used to knock out defective genes, insert new genes into cells, and tackle genetic diseases such as sickle cell anemia and cystic fibrosis. Furthermore, it can target genes responsible for neurodegenerative diseases like Alzheimer’s and Huntington’s. Lastly, it can create cells resistant to viral infections such as HIV and Hepatitis B (Li et al., 2020). CRISPR-based GED techniques have evolved to encompass base editing (BED) (Gaudelli et al., 2017), prime editing (PED) (Anzalone et al., 2019), and epigenome editing (epi-GED) (Goell and Hilton, 2021). Each of these methods offers distinct benefits and drawbacks and can be valuable in specific circumstances. There is a need for interventions and decisions at multiple levels, as illustrated in Figure 1 and Figure 2. This emphasizes the importance of AI in the process of making appropriate choices that are specifically tailored to address distinct situations in the genome editing process.

Using AI in GED is imperative and holds the promise to revolutionize the healthcare sector. CRISPR-based editing technologies like CRISPR/Cas9 allow for precise and targeted editing of the genetic code of organisms, which is a major breakthrough in biotechnology (Tyagi et al., 2020). However, AI’s integration with CRISPR, improves the overall GED pipeline, providing new insights, capabilities, and opportunities for manipulating and understanding the genetic code. The recent advances in multi-omics technologies that can produce big data from different sources, such as genes, RNA, proteins, and DNA modifications, have made AI necessary for analyzing medical information (Hamet and Tremblay, 2017). Deep learning (DL) and Machine Learning (ML) models have been used to analyze and comprehend large and complex genomic data sets (Quazi, 2022). These studies could prove valuable in identifying more appropriate features for AI models, thereby enhancing their ability to predict editing outcomes such as off-target editing. For example, in cancer, AI models can utilize genomic data to identify cancer subtypes, and CRISPR-based GED can assist in engineering immune cells capable of targeting these subtypes or disrupting oncogenes (Katti et al., 2022). Leenay et al. (2019) developed an ML algorithm called SPROUT that can predict the repair outcomes of GED in primary T cells with high accuracy. SPROUT was trained on a large dataset of CRISPR-Cas9 editing events, and it can be used to design CRISPR experiments to maximize the desired editing outcome. It is a valuable tool for researchers who are using CRISPR-Cas9 to develop new therapies for cancer and other diseases. CRISPR technology is advancing quickly. As shown in Figure 1, Cas9 is not the only option for GED. There are other variants of Cas proteins that are being investigated for this purpose. Some examples are CRISPR-Cas12 (Xiao et al., 2021; Senthilnathan et al., 2023), CRISPR-Cas13 (Kavuri et al., 2022), CRISPR-Cas3 (Morisaka et al., 2019), and many others. Therefore, the role of AI approaches should become more important. The complete list of abbreviations and their full forms used in this paper is provided in Table 1.

**TABLE 1:** A list of all the acronyms and their full names used in this article.

Acronym	Definition
ABEs	Adenine base editors
AI	Artificial Intelligence
BED	Base editing
Bi-LSTM	Bidirectional long short-term
CBEs	Cytosine base editors
CRISPR	Clustered regularly interspaced short palindromic repeat
CNN	Convolutional neural network
dCas9	Dead Cas9
DL	Deep learning
DNA	Deoxyribonucleic Acid
epi-GED	Epigenome editing
gRNAs	Guide RNAs
GED	Genome editing
HDAC3	Histone Deacetylase 3
indels	Insertion or deletion of small fragments
KRAB	Krüppel-associated box
LR	Linear regression
MMEJ	Microhomology-mediated end-joining
ML	Machine learning
mRNA	Messenger Ribonucleic Acid
nCas9	Nickase Cas9
ngRNA	Nick Guide RNA
NHEJ	Nonhomologous end-joining
PED	Prime editing
RNN	Recurrent Neural Network
RT	Reverse Transcriptase
sgRNA	single-guide RNA
SNVs	Single Nucleotide Variant
SVM	Support vector machine
TET3	Tet methylcytosine dioxygenase 3
TALENs	Transcription activator-like effector nucleases
ZFNs	Zinc-finger nucleases

The review on “Advancing Genome Editing with AI: Opportunities, Challenges, and Future Directions” highlights several key contribution points. It emphasizes the critical role of AI in advancing GED, especially in the context of CRISPR-based technologies. It underscores how AI enhances the precision, efficiency, and cost-effectiveness of gene editing, making it a powerful tool in addressing a broad range of human diseases. One of the key contributions is the discussion of AI models used for designing guide RNAs (gRNAs) in CRISPR-based GED. It explains how AI models, including ML and DL, are employed to predict gRNA efficiency, offering remarkable accuracy in identifying optimal gRNAs for specific applications. The review delves into the role of AI in improving BED, PED, and epi-GED techniques. It describes how AI models have been developed to predict base efficiency and editing patterns with high accuracy, thus facilitating the correction of genetic mutations. The review points out the potential of AI, CRISPR, and precision medicine in personalizing treatments based on individual genetic profiles. AI is depicted as a critical component for analyzing patient data and suggesting specific gene modifications to tailor treatments to individual patients, ensuring therapies are more precise and effective. It also contributes to the field by providing a comprehensive overview of the synergistic relationship between AI and GED. It showcases the transformative potential of this collaboration and its implications for healthcare, biomedicine, and genetics.

### 1.1 Research methodology and the literature sources

This study offers insights from various online databases such as PubMed Central, Scopus, Medline, and Google Scholar. It compiles information from studies and research findings that explore the utilization of ML and DL approaches for genome editing technologies. Table 2 displays the keywords used for the database searches. Additionally, this assessment examined the work of other academics and made fresh research recommendations.

**TABLE 2:** Queries made using specific keywords in databases.

Search term	Set of keywords
Editing	Genome editing, CRISPR/Cas9 editing, Base editing, Prime editing, Epigenome editing
Deep	Deep learning, Deep neural network
Machine	Machine learning
AI	AI in genome editing, AI in gRNA design
Deep learning techniques	Recurrent neural networks, Deep autoencoders, long short-term memory, Deep neural network, deep belief network, deep convolutional neural network, deep Boltzmann machine, deep reinforcement learning, extreme learning machine
Machine learning techniques	Artificial neural network, naïve Bayes, decision tree, k-nearest neighbors, k-means clustering, random forest, support vector machines, ensemble learning

#### 1.1.1 Inclusion criteria

The inclusion of articles in the review was based on their eligibility and the distinctiveness of the topic. The selection was confined to papers published in English. Furthermore, this assessment did not consider case studies, comments, or letters to the editor.

#### 1.1.2 Elimination criteria

The first level of exclusion involved the evaluation of abstracts. The subsequent steps included data extraction and a thorough analysis of the full texts. The articles were then disregarded due to their lack of relevance, English language proficiency, or bad writing.

#### 1.1.3 Results

**FIGURE 3:**
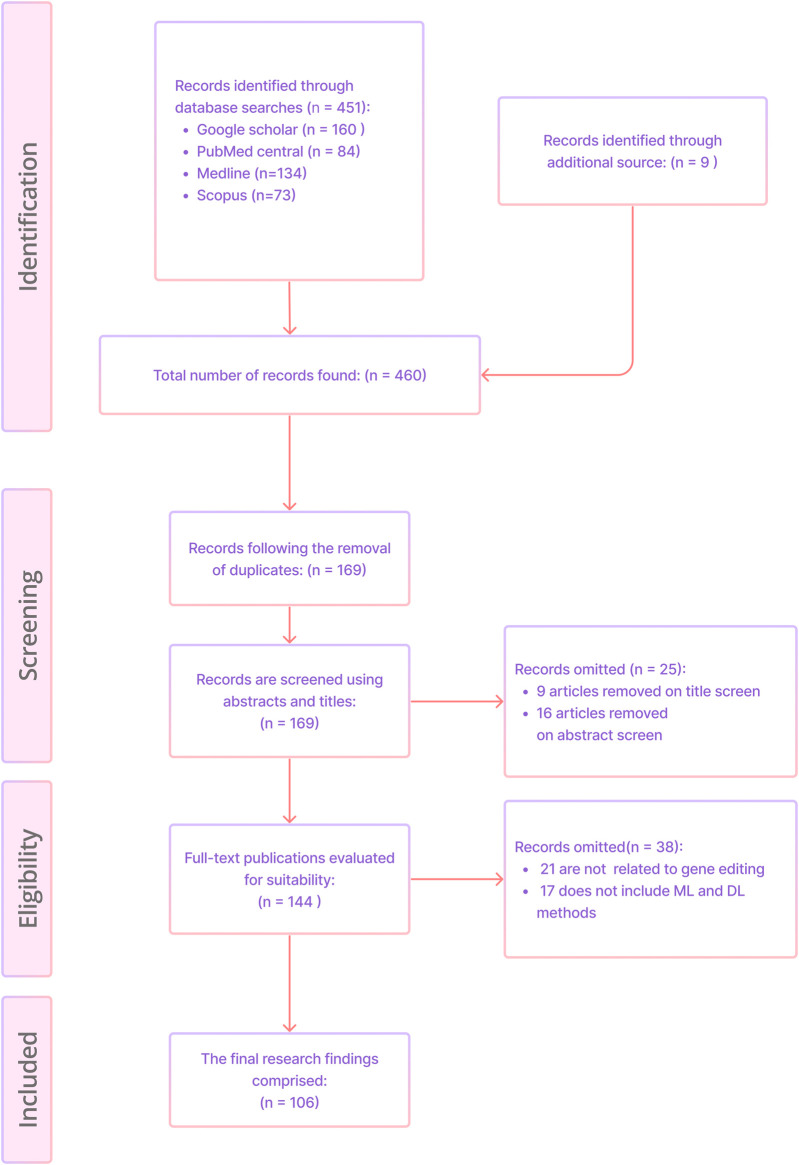
Article selection using the PRISMA ScR methodology.

A total of 460 unique publications were acquired from PubMed Central, Scopus, Medline and Google Scholar, and other sources. After screening the titles and abstracts, 25 papers were excluded. Additionally 38 articles were removed based on full text analysis, leaving behind 106 articles for final assessment. Figure 3 shows the article selection using the PRISMA methodology.

## 2 Understanding AI

AI is a branch of computer science that focuses on creating systems or machines capable of performing tasks that typically require human intelligence. AI, particularly ML and DL, has emerged as a transformative force, revolutionizing the way we approach diagnostics, treatment, and even gene editing. When it comes to gene editing, AI models play a pivotal role in deciphering complex genetic information, identifying patterns, and predicting potential outcomes. The creation of these models involves a multi-faceted process that leverages both ML and DL techniques.

### 2.1 Data collection and preprocessing

The first step in developing AI models for gene editing involves the collection of extensive genetic data. This data may include information from various sources, such as genomic sequencing, patient records, and experimental results. Once collected, the data undergoes thorough preprocessing to ensure its quality and relevance. This step is crucial to remove noise and irrelevant information, allowing the AI models to focus on meaningful patterns.

### 2.2 Model selection

Choosing the right type of model is essential for the success of AI applications in gene editing. In this context, both traditional ML algorithms and sophisticated DL architectures are considered. ML models, like decision trees or support vector machines, may be employed for simpler tasks, while DL models, especially deep neural networks, are preferred for handling the intricate relationships within complex genetic data.

### 2.3 Training the model

Training the AI model involves exposing it to a labeled dataset where it can learn the patterns and relationships within the genetic information. Supervised learning techniques are often employed, where the model is trained on examples with known outcomes. The model adjusts its parameters iteratively until it can accurately predict outcomes based on new, unseen data.

### 2.4 Optimization

Once the model is trained, optimization is performed to enhance its performance. This involves fine-tuning parameters, adjusting architectures, and employing optimization algorithms to maximize the accuracy and efficiency of the gene editing predictions. Continuous feedback loops may be established to update the model as more data becomes available or as our understanding of genetic processes evolves.

### 2.5 Integration into healthcare systems

The finalized AI models are integrated into healthcare systems to assist clinicians in making informed decisions regarding gene editing. These models can provide insights into potential genetic disorders, identify optimal gene editing strategies, and predict patient responses to specific interventions.

**FIGURE 4:**
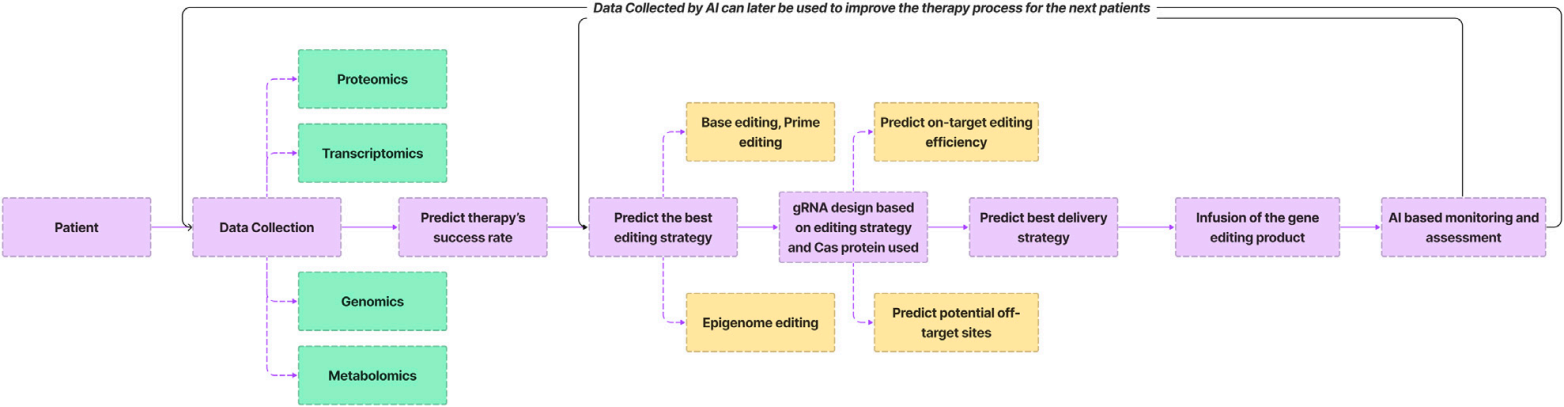
AI driven gene therapy process.

AI holds significant promise in optimizing various facets of the genome editing process (Figure 4). The collection of multi omics data from individuals undergoing gene therapy provides a rich dataset that can be leveraged by AI algorithms. Through the analysis of this data, AI can forecast the likelihood of successful gene editing outcomes for specific patients based on patterns observed in previous cases. Moreover, AI plays a pivotal role in guiding the genome editing process by aiding in the selection of optimal editing strategies. This includes the design of guide RNAs (gRNA) with heightened precision—minimizing off-target effects while maximizing on-target editing efficiency. AI algorithms also contribute to the identification of the most suitable delivery strategies for the genome editor. In the post-infusion phase, it facilitates real-time monitoring and assessment of patients, enabling a dynamic evaluation of therapeutic efficacy and potential complications. Subsequent iterations of the workflow benefit from AI-driven enhancements, refining predictions and strategies for future patients.

## 3 AI in gRNA design for CRISPR/Cas-based genome editing

**FIGURE 5:**
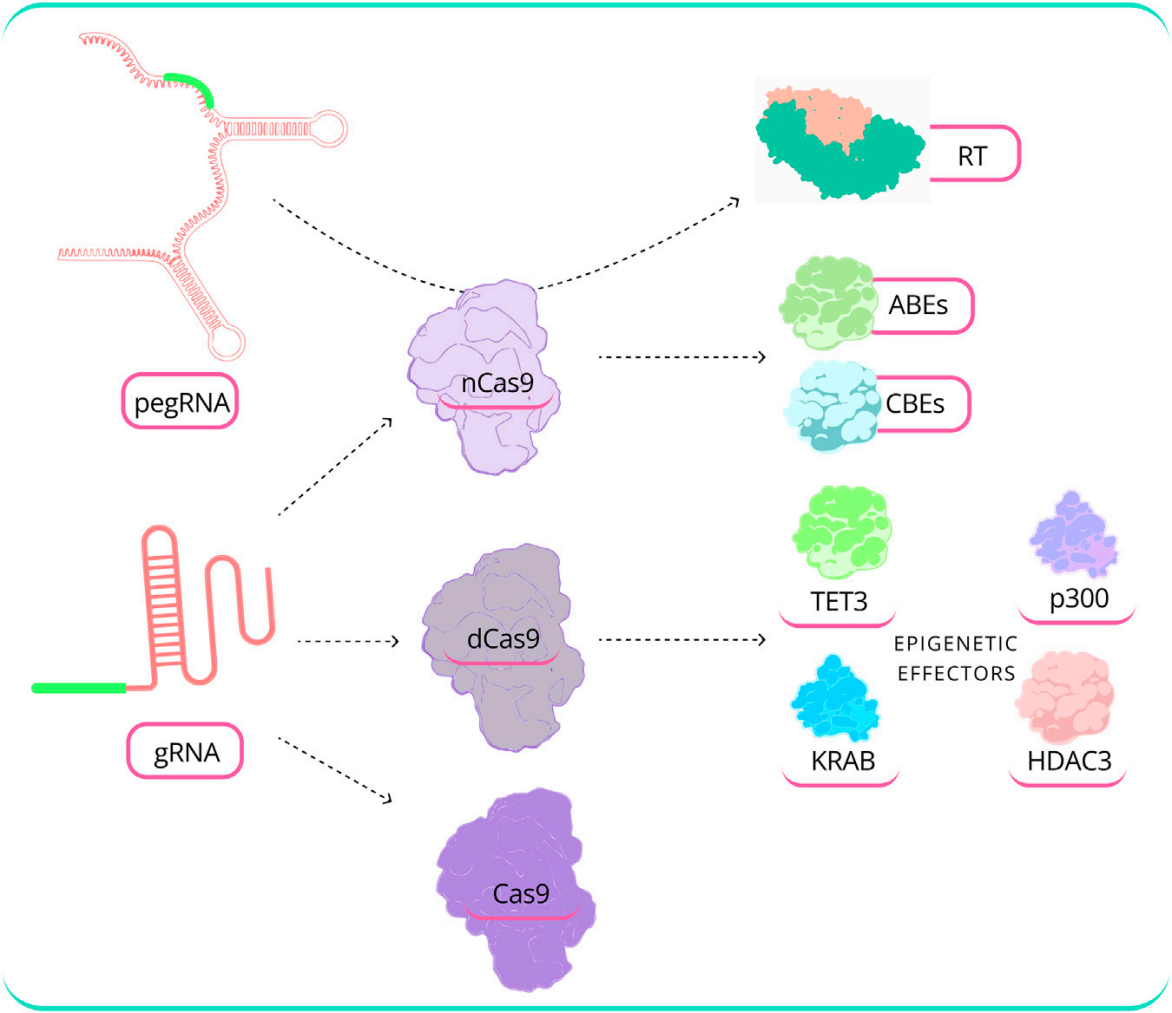
Modular nature of CRISPR-based editing technologies. CRISPR/Cas technology consists of various elements, and the complexity grows in advanced editing methods, such as epigenome editing, where the selection of the effector and the appropriate effector combination varies according to specific needs. [ TET3: Tet methylcytosine dioxygenase 3, KRAB: Krüppel-associated box, HDAC3: Histone Deacetylase 3, RT: Reverse Transcriptase, dCas9: Dead Cas9, nCas9: Nickase Cas9, ABEs: Adenine base editors, CBEs: Cytosine base editor (Abbreviation: Table 1)].

The effectiveness of GED relies on the selection of the gRNA sequence. Certain gRNAs have the capacity to disrupt nearly all target alleles within a cell population, while others exhibit minimal or no observable activity (Lee et al., 2018). Consequently, a range of gRNA design tools have been developed, primarily employing ML and DL algorithms to address this challenge. Figure 5 illustrates the modular nature of CRISPR-based editing technologies.

Several ML and DL models have been developed for predicting the efficiency of gRNAs. In accordance with prior research, the selection of an appropriate methodology holds paramount importance in the development of reliable models for predicting gRNA efficiency (on-target/off-target binding) (Doench et al., 2016). While Linear Regression (LR) has shown a certain level of effectiveness (Moreno-Mateos et al., 2015), more efficient models utilize advanced methods like Support Vector Machines (SVM) (Chari et al., 2015; Wong et al., 2015) and Gradient Boosted Trees (GBT) (Doench et al., 2016). These advanced techniques are particularly advantageous as they incorporate considerations of intricate feature interactions (McKinney et al., 2006). Several tools were developed to predict the efficiency of these guides. Wessels et al. (Wessels et al., 2023) developed a CNN named TIGER to forecast efficacy based on guide RNA sequence and context. Almost 200,000 RfxCas13d guide RNAs were created and tested that deliberately included designed mismatches, insertions, and deletions (indels) to target crucial genes in human cells. It was observed that utilizing gRNA efficacy estimates from the TIGER combination model could effectively distinguish between vital genes and control genes. TIGER outperformed the present AI models in predicting on-target/off-target activity. Xiang et al. (2021) developed a DL model called CRISPRon that can predict the efficiency of gRNAs with high accuracy. A dataset of on-target gRNA activity combined with additional published data was created, and the CRISPRon model was trained on 23,902 gRNAs. The model’s primary constraint arises from the fact that the double-strand breaks (DSBs) induced by Cas9 undergo repair through MMEJ and NHEJ pathways. Consequently, this repair process results in small indels at the site of the DSB or large translocations. There are several other DL-based models like DeepHF, developed by Wang et al. (2019) that outperformed other gRNA design tools for highly specific Cas9 variants. Zhang et al. (2021) proposed attention-based CNNs, CRISPR-ONT (T: Target) and CRISPR-OFFT, for predicting on-target and off-target activities of sgRNAs. Xue et al. (2018) introduced DeepCas9, a DL framework based on CNN, which accurately predicts functional sgRNAs for the CRISPR-Cas9 system.

Recently, there has been ongoing progress in the development of off-target prediction algorithms. These algorithms are typically trained using data obtained from *in vitro* cleavage assays, which involve using immortalized cell lines (Störtz and Minary, 2021). Immortalized cell lines provide a controlled environment for conducting experiments related to GED (Mehravar et al., 2019), which makes them a valuable resource for training predictive models. It has been observed that these off-target prediction algorithms tend to rely heavily on sequence-based information, which demonstrates a strong connection with the actual cleavage activity of CRISPR/Cas9 or similar gene-editing systems. Physical attributes of the genome such as chromatin accessibility and DNA methylation pattern features, currently underutilized in AI models, provide valuable insights into the three-dimensional structure and packaging of DNA in the cell, which can impact the accessibility of specific genomic regions for gene editing. In their study, Störtz et al. (2023) developed a method known as piCRISPR, which considers a combination of sequence-based attributes and physically informed features, including factors like chromatin accessibility and DNA methylation. Through an extensive assessment using a substantial dataset of CRISPR/Cas9 editing occurrences, piCRISPR exhibited superior performance compared to all other existing prediction methods for off-target cleavage activity. The CNN models yielded the best performance benchmarks, with CNN S5E2 achieving the highest accuracy (AU - ROC = 0.998). piCRISPR can also effectively pinpoint new off-target cleavage sites and facilitate the design of CRISPR/Cas9 experiments with reduced off-target cleavage potential.

Exon skipping is a promising therapeutic approach for genetic diseases caused by mutations in exons. CRISPR/Cas9 GED can be used to induce exon skipping by targeting the splice acceptor or donor sites of the target exon. SkipGuide (Louie et al., 2021) is a tool that helps design CRISPR/Cas9-based therapies for genetic diseases by inducing exon skipping. It tested over 1,000 guide RNAs on 791 splice sequences in mouse cells and predicted the exon-skipping frequencies with high accuracy. SkipGuide can save time and resources by finding effective guide RNAs for exon skipping. The precise selection of guide RNA sequences is crucial for successful GED, and various ML and DL models have been developed to predict the efficiency of these sequences. These advanced techniques, such as SVMs, GBTs, and CNN-based models like TIGER and CRISPRon, have shown remarkable accuracy in predicting on-target and off-target activities, making them valuable tools in designing and optimizing CRISPR-based therapies. These advancements mark a significant leap toward harnessing the full potential of CRISPR/Cas technology for precise and effective GED.

## 4 Role of AI in enhancing advanced genome editing pipelines

AI can be used to enhance advanced GED pipelines by providing tools and methods for designing, optimizing, and evaluating GED experiments. AI-driven models and tools are instrumental in enhancing the precision, efficiency, and cost-effectiveness of GED techniques, making them a robust tool for addressing a wide range of human diseases. Table 3 provides a comprehensive list of databases that are associated with GED research, serving as valuable resources for the development of AI models. These databases offer a wealth of information, ranging from genetic sequences and variations to experimentally verified data on GED outcomes. Table 4, on the other hand, compiles ML and DL-based tools specifically designed for various GED applications. AI-powered models and tools play crucial roles within sophisticated genome editing pipelines. Their contributions can be classified into the following categories: tools for designing gRNA to predict on-target and off-target editing, and tools specifically developed for predicting outcomes in advanced genome techniques.

**TABLE 3:** Databases associated with genome editing research for the development of AI models.

Dataset name	Data description	Data link	Type of editing	Target	Machine learning model used
CHANGE-seq data Lazzarotto et al. (2020), (publicly available)	There are a total of 201,934 off-target sites scattered throughout the human genome	https://github.com/tsailabSJ/changeseq	CRISPR-Cas9 genome-editing	Off-target	GBT
DeepHf data Wang et al. (2019), (publicly available)	For each nuclease, there are 50,000 gRNAs available, collectively targeting approximately 20,000 genes	http://www.deephf.com/	CRISPR-Cas9 GED	On-target	RNN (Recurrent Neural Network), Bi-LSTM (bidirectional long short-term)
Abadi et al. (2017), (publicly available)	This comprises 33 sets of sgRNAs, each associated with its specific targets	https://doi.org/10.1371/journal.pcbi.1005807.s014	CRISPR-Cas9 GED	Off-targets	Random forest
Genome CRISPR database Rauscher et al. (2017), (publicly available)	There is a total of 400,000 sgRNA sequences from the GenomeCRISPR project dataset.	http://genomecrispr.org/	CRISPR-Cas9 GED	On-targets	CNN (named as DeepSgRNA)
GUIDE-seq data Tsai et al. (2015), (publicly available)	Nucleases guided by RNA from two human cell lines, U2OS and HEK293, were examined at different sites	https://github.com/tsailabSJ/guideseq	CRISPR-Cas9 genome-editing	Off-targets	CRISTA (CRISPR Target Assessment using RF regression)
Arbab et al. (2020), (publicly available)	Data from as many as 10,638 sgRNA-target pairs was randomly divided into partitions	https://www.google.com/url?q=https://ars.els-cdn.com/content/image/1-s2.0-S0092867420306322-mmc5.csv&sa=D&source=docs&ust=1698141109170457&usg=AOvVaw1eJY32CwBjGjzLD64EITS8	BED	On-target	Be-Hive (autoregressive neural network)
Kim et al. (2023), (publicly available)	Nine Cas9 variants	https://www.ncbi.nlm.nih.gov/bioproject/PRJNA821929/	BED	On and Off-target	SVM, L1-regularized LR, L2-regularized LR, AdaBoost, and Random Forest
Pallaseni et al. (2022), (publicly available)	Used the dataset from (Arbab et al., 2020; Song et al., 2020)	https://www.ebi.ac.uk/ena/browser/home	BED	Off-target	GBT
Li et al. (2022), Private	1134 target sequences	https://www.ncbi.nlm.nih.gov/bioproject/PRJNA885770/	BED	On-target	XGBoost
Kim et al. (2021), (publicly available)	There are 54,836 pairs consisting of pegRNAs and their corresponding target sequences	https://github.com/julianeweller/MinsePIE	PED	On and Off-target	DeepPE

**TABLE 4:** ML and DL-based tools for Genome editing applications.

Ref	ML models used	Dataset	Description and key contribution	Performance evaluation metrics	Limitation	Target
Chuai et al. (2018)	DeepCRISPR (DCDNN)	Thirteen distinct human cell lines produced a total of 0.68 billion sgRNA sequences	This computational framework surpasses existing *in silico* tools by combining sgRNA on-target/off-target site prediction into a single system with DL.	Spearman: 0.246, AUROC: 0.804, AUPRC: 0.303	The model acquires an understanding of which attributes are crucial for improved sgRNA structure, even when trained with a limited number of samples	On and Off-target
Lin and Wong (2018)	CNN and FNN, Random Forest, GBTs, and LR	GUIDE-seq Tsai et al. (2015), CRISPOR Concordet and Haeussler (2018)	The key contribution of this paper is the development and implementation of a deep CNN for accurately predicting off-target mutations in CRISPR-Cas9 gene editing	AUROC: 97.2% for CNN, AUROC: 97% for FNN	—	Off-target
Xue et al. (2018)	DeepCas9 (1D CNN)	Wang Wang et al. (2014), Doench V1 Doench et al. (2014), Doench V2 Doench et al. (2016), C.elegans (F et al. (2015) HCT116 Hart et al. (2015), Z fish Gagnon et al. (2014); Moreno-Mateos et al. (2015); Varshney et al. (2015), Chari Chari et al. (2015), Haeussler Haeussler et al. (2016), HL-60 Xu et al. (2015)	It is the first DL technique that can recognize CRISPRCas9 sgRNA activity directly from genetic sequences without the need for feature input	Spearman: 0.23-0.61	These datasets’ sgRNA activity was completely limited to clinical assays, where the measured cleavage efficiency served as a clear indicator of KO efficacy	On-target
Liu et al. (2019)	SeqCrispr (RNN + CNN + transfer learning)	DeepCRISPR Chuai et al. (2018), CRISPR-Cpf1 Zaidi et al. (2017)	SeqCrispr is a DL model, which integrates gene network features specific to a given context into the model	Spearman: 0.77	The limited knowledge of gene activity and its fluctuating effects on phenotype, and the challenging biological interpretation of computational models all restrict the predictive model’s efficiency	On-target
Wang et al. (2019)	DeepHF (RNN)	With approximately 50,000 gRNAs, DeepHF is the biggest gRNA on-target activity set for cells from mammals	To create the final model, DeepHF extracts features using a Bi-LSTM and combines them with biological features that are manually created. Important sequence characteristics linked to gRNA activity were found in the study, which also assessed several ML algorithms for gRNA activity prediction	Spearman: 0.867, 0.862, and 0.860	They were unable to determine which algorithm performed more effectively than others on endogenous sites because of the small amount of data available	On-target
Shrawgi and Sisodia (2019)	DeepSgRNA (CNN, with Hierarchical feature generation abilities)	40,000 sgRNA sequence examples taken from the GenomeCRISPR project database	DeepSgRNA finds and forecasts RNA guides to improve performance. There is no need to create any features with the suggested model	Spearman: 0.82, AUROC: 0.85	Specific sgRNA’s off-site effects have not been considered in this investigation	On-target
Wang and Zhang (2019)	CNN with 5layers + transfer learning	Cas9, eSpCas9, Cas9 (/\recA) Yue et al. (2020)	The main contribution of this paper is the development of a CNN_5 layers network for predicting sgRNA activity in prokaryotic and eukaryotic species. The model takes 43nt-long DNA sequences as input and predicts on-target activity	Spearman: 0.582, 0.7105, 0.360	The limitation of this model is that it does not perform well in predicting the on-target activity for the Cas9 (/\recA) scenario	On-target
Aktas et al. (2019)	CNN, MLP, Bi-LSTM	DeepCRISPR Chuai et al. (2018)	In this work, sgRNA target estimate for CRISPR/CAS9 with DL was carried out to reduce these genomic aberrations	Accuracy: 96.7%	Some of the mistargeted positions caused unwanted genome distortions	Off-target and on-target
Kim et al. (2019)	DeepSpCas9 (3 1D-CNN)	DeepSpCas9	It accurately predicted the activity of the SpCas9 enzyme	Spearman: 0.73	The size of the training datasets was not ideal	On-target
Liu et al. (2020)	CnnCrispr (Bi-LSTM and CNN)	DeepCRISPR Chuai et al. (2018)	To forecast the off-target tendency of sgRNA at particular DNA fragments, CnnCrispr was proposed	AUROC: 0.957, AUPRC: 0.429	RNNs are capable of implementing memory functions, but their capacity is restricted due to the possibility of gradient explosion or disappearance	Off-target

### 4.1 Base editing and AI

Base editing is a powerful GED method that allows precise conversion of individual genomic nucleotides with high efficiency without requiring double-stranded breaks (Rees and Liu, 2018). In their work, Evanoff et al. (Evanoff and Komor, 2019) present a comprehensive summary of base editors, highlighting their modular design (Figure 5) and the range of options for every part. Several ML and DL models have been created with the aim of enhancing the efficiency of base editors with a primary focus on improving editing outcomes. Arbab et al. (2020) developed BE-Hive, an ML model that uses a deep conditional autoregressive model to predict editing sequences and base effectiveness. The model achieved high accuracy in predicting BED genotypic outcomes and efficiency. BE-Hive was later used to design BED strategies for correcting many SNVs linked to the disease with ≥90% accuracy, some containing bystander nucleic acids.

Pallaseni et al. (2022) developed an ML model to predict the efficiency and patterns of BED. The model used sequence features and position information to predict editing outcomes for different types of base editors. The model showed better generalization across various datasets with a precision varied from 0.49 to 0.72 among editors. A significant finding from the research pertains to BED, highlighting that its efficacy is influenced by the sequence, particularly with the most pronounced impact originating from the nucleotides surrounding the target base. This makes it difficult to predict whether a particular target will be edited efficiently, and how many bystander mutations will be introduced. To overcome this, Song et al. (2020) trained a DL model that considered both sequence-based features and the positions of the target nucleotides within the genome. The model outperformed existing models in the efficiency prediction of ABEs and CBEs with high accuracy, exhibiting Pearson correlation values between 0.50 and 0.95. Predicting the outcomes of BED has been a widely explored subject in this domain, with DL being the predominant approach used in most instances. Marquart et al. (2021) developed BE-DICT, a DL model that predicted the outcomes of adenine- and cytosine-based editors using an attention-based algorithm. It uses a protospacer sequence as input and calculates the editing probability as an output for every target nucleotide. This model was trained on different datasets and found different AUCs: ABEmax: 0.86, CBE4max: 0.94, ABE8e: 0.66, and TargetAID: 0.97. Park and Kim (2023) present two DL models, DeepABE and DeepCBE, available as web tools, for predicting the BED efficiencies and outcomes of ABEs and CBEs, respectively. Kim et al. (2023) assessed nine Cas9 variants, each designed to recognize distinct PAM (Protospacer Adjacent Motif) sequences. A DL model called DeepCas9variants was designed to predict the most effective Cas9 variant for targeting specific sites based on the intended patterns. Later a computational model called DeepBE was also developed to forecast the editing efficiency and productivity of 63 base editors. It was discovered that the Pearson and Spearman correlation coefficients ranged from 0.82 to 0.95 and 0.80 to 0.94, respectively. Li et al. (2022) developed CAELM, an ML model that predicts the efficiency of cytosine base editors (CBEs) using chromatin reachability and sequence context. CAELM was shown to accurately forecast the outcome of *in-situ* BED. They previously used CNN (Feng et al., 2019) to predict the efficiency of GBE base editing, but in this study, they chose the XGB Regressor because their dataset only included the editing results for 1134 target patterns, and the XGB Regressor frequently performed better than DNNs when working with small datasets (Fernoaga et al., 2020). The model’s accuracy was evaluated using Pearson’s correlation value, which yielded a r value of 0.64 within the predicted and measured values. Chen et al. (2022) created CGBEs (C•G•toG•C base editors) with a variety of editing profiles. In mammalian cells, on a collection of 10,638 genomically merged target locations, they described ten promising CGBEs. Using this information (Koblan et al., 2021), developed ML models that correctly predicted the quality and yield of editing results (R = 0.90). These CGBEs allow for the >90% precise and up to 70% efficient repair of 546 transversion single-nucleotide mutations linked to diseases that affect the wild-type coding patterns. AI-supported structural predictions like AlphaFold2 or diffusion models can be employed to develop better variants of base editors. Huang et al. (2023) used AlphaFold2 to develop new cytosine base editors with distinct features. AlphaFold2 is a tool for predicting protein structures with remarkable accuracy. However, it has its limitations when dealing with proteins that share a very high degree of sequence similarity. When proteins have sequences that are nearly identical, AlphaFold2 may struggle to differentiate between them, and it might face challenges in accurately characterizing structural differences or functional distinctions that arise from Single Nucleotide Polymorphisms (SNPs). The role of AI in advancing BED technologies is undeniably transformative. AI-driven predictions have greatly enhanced our ability to design more efficient and precise base editors, significantly impacting GED and potential therapeutic applications. Moreover, AI’s potential is not limited to sequence-based predictions alone; it extends to structural innovations as well. The integration of AI, exemplified by tools like AlphaFold2, allows us to venture into the development of novel base editors with distinct features, further illustrating the profound impact AI has in shaping the future of genetic medicine and GED.

### 4.2 Prime editing and AI

Prime editing is an emerging technique that utilizes reverse transcription to insert programmed sequence modifications into DNA sequences (Yan et al., 2020). It is an adaptable GED tool, capable of making a wide range of genetic changes, but achieving high editing efficiency and product purity necessitates PED guide RNA (pegRNA) experimental optimization (Mathis et al., 2023). It consists of three main components: a reverse transcriptase, pegRNA, and a Cas9 nickase. The pegRNA contains both the target sequence and the edit sequence, which are used to direct the desired modification in the DNA. Creating pegRNAs presents a greater challenge compared to designing guides for other CRISPR-based editing methods. Fortunately, tools such as Easy-Prime (Li et al., 2021) and PrimeDesign (Hsu et al., 2021) are available to assist in this complex design process. Easy-Prime was created by Li et al. (Li et al., 2021) and trained on previously released PED datasets. To prioritize pegRNA candidates and forecast their editing effectiveness, Easy-Prime makes use of well-known and recently discovered traits, as well as projected RNA folding and secondary structure. They have shown optimization of prime editor guides for correcting mutations in 89.5% of the 152,351 Genome-Wide Association Studies (GWAS) variants. Easy-Prime can also generate optimum pegRNAs for many genetic variations associated with diseases. PrimeDesign is another tool for pegRNA design, developed by Hsu et al. (2021), designed for a number of different editing tasks, containing single nucleotide substitutions, additions, deletions, and inversions. To address these harmful alleles, they created potential pegRNAs and ngRNAs using harmful human genetic variations according to ClinVar8 (*n* = 69,481). They discovered that 91.7% of these pathogenic variants are susceptible to targeting preferably by one pegRNA spacer with 34 replica maximal length nucleotides. They tested the pegRNAs and ngRNAs that PrimeDesign created to make different modifications, and they found that not every design produced the intended adjustments at elevated frequencies. As a result, users of PrimeDesign might still be required to modify their pegRNA selections after evaluating the original suggestions.

Several ML and DL tools have been developed for predicting the outcome of PED. Koeppel et al. (2023) investigated the factors influencing the efficiency of PED insertions. Based on their findings, they developed an ML model to predict PED insertion efficiencies. The model considers the nucleic acid’s structure, length, and the insertion sequence’s secondary structure, as well as the expression levels of TREX1 and TREX2. This is because TREX1 and TREX2 degrade the 3′ flap of DNA, which is necessary for PED insertions. An ML model was also developed to predicts the insertion efficiency of the PED technique. The model uses sequence features, including the length and composition of the insert sequence, in addition to the flanking DNA sequence and DNA repair proteins as inputs. The model was trained and tested on different sequences, locations, and human cell lines. The model found that the insertion rate depends on the sequence length, composition, and structure. Mathis et al. (2023) created PREDICT, a DL model that predicts the outcomes and rates of PEDs. It uses an RNN to learn from a large dataset of over 90,000 PED experiments. With a Spearman’s R for planned and accidental edits of 0.85 and 0.78, respectively, PREDICT accurately predicts editing rates for all small-sized genomic alterations. PED offers versatile genetic modifications, including base changes, insertions, and deletions, and holds promise for rectifying disease-related human mutations. Its efficiency relies on factors like the target and edit sequences, along with the DNA mismatch repair pathway. Current research predominantly focuses on augmenting AI models with novel factors to improve prediction accuracy, exemplified by discoveries such as TREX1 and TREX2.

### 4.3 Epigenome editing and AI

Unlike traditional gene editing, which focuses on altering the genetic code, epi-GED allows for targeted modifications in the way genes are regulated, turned on or off, without changing the DNA sequence itself. It allows the manipulation of DNA methylation patterns, histone modification, and RNA editing, to alter gene expression. It has potential applications in disease treatment, functional genomics research, and stem cell therapies. By using epi-GED, researchers and clinicians can target specific genes or pathways that are involved in various diseases or cellular functions, and modulate their expression.

CRISPR/Cas-based epi-GED is a powerful technique that can be employed to regulate gene expression without changing the DNA sequence. This is achieved by targeting specific DNA sequences with CRISPR/Cas nucleases and fusing them with epigenetic modifiers (Goell and Hilton, 2021). Rauschert et al. (2020) and Machnicka and Wilczynski (2020) discuss the application of ML and DL methods in analyzing epigenomic data, which can aid in understanding epigenetic mechanisms and reconstructing the epigenetic code. Epigenome editing utilizes a completely distinct approach to gene regulation when compared to other CRISPR-based editing techniques. Researchers have recognized this distinction and have initiated the development of dedicated AI tools to meet the requirements of epi-GED. EpiCas-DL (Yang et al., 2023) is a tool that uses DL to predict the activity of sgRNAs for CRISPR-mediated epi-GED. It incorporates four types of epigenetic features, including gene expression, methylation, chromatin accessibility, and the separation between the transcription start site and the target site, to enhance prediction accuracy. EpiCas-DL outperforms other existing methods with an AUC of 0.87 and also identifies the key factors that influence the effectiveness of sgRNA in activating and silencing genes. It can be utilized to enhance the sgRNA design for gene regulation without altering the DNA sequence. The application of AI algorithms in the field of epi-GED is still an emerging and relatively unexplored area when compared to base and prime editing. One reason for this could be the dynamic nature of epigenomic data, which includes DNA methylation patterns, histone modifications, and chromatin accessibility.

## 5 AI, CRISPR, and precision medicine

Precision medicine involves personalizing medicine to tailor treatment based on biological or molecular profiling, for a particular population or even a single patient. This might be achieved using the information pertaining to the genome, transcriptome, epigenome, or proteome. CRISPR-Cas9 enables precise and efficient editing of the human genome, which can be utilized to fix mutations that cause tumors, disable oncogenes, or activate tumor suppressor genes (Das et al., 2022). For instance, CRISPR-Cas9 could be employed to develop new genetic tests for identifying individuals at risk of developing certain diseases. It could also be used to create novel gene-editing therapies for treating genetic disorders and cancer (Semiz and Aka, 2019). The convergence of AI, CRISPR gene editing, and precision medicine represents a transformative frontier in healthcare and biomedical research. By harnessing AI’s data analysis and predictive capabilities, gene editing techniques like CRISPR can become more precise and effective in altering genes responsible for various diseases. AI-driven genomic analysis helps in identifying genetic variations associated with diseases or patient’s response to a particular treatment. CRISPR can be used to modify these genes, either to correct mutations or enhance the patient’s response to treatment, taking into account their genetic makeup. ML-based tools, like AlphaMissense, can predict the pathogenicity of missense variants in human proteins with high accuracy (Cheng et al., 2023). In another study, Sundaram et al. (2018) exhibited that deep neural networks could be applied to determine new candidate genes for rare diseases. CRISPR-based genetic modification can then be employed to correct these disease mutations (Cai et al., 2016). Genetic profiling through CRISPR (Bock et al., 2022) and AI can help in identifying an individual’s predisposition to certain diseases, enabling early intervention and preventive measures. Precision medicine, focusing on personalizing treatment based on genetic and molecular profiling, holds the promise of more effective and targeted medical interventions. CRISPR-Cas9, with its precise GED capabilities, provides the opportunity to fix mutations that cause disease and enable the development of innovative diagnostic tests and therapies. Moreover, ML-based tools, such as AlphaMissense and deep neural networks, exhibit high accuracy in assessing genetic variants and identifying candidate genes for rare diseases, offering invaluable support for CRISPR-mediated gene editing.

## 6 Open challenges

The two major challenges in CRISPR technology are high costs and the need for more efficient GED processes. For instance, the approval of Hemgenix gene therapy for Haemophilia B costs a staggering $3.5 million per treatment, making it the most expensive medication worldwide (Naddaf, 2022). AI may be able to help with these issues by assisting in the selection of optimal genetic sequences and experimental protocols, reducing trial-and-error efforts, and improving predictive accuracy. Furthermore, AI can aid in streamlining clinical trials and optimizing supply chains, ultimately leading to more cost-effective treatments. For instance, in the planning of clinical trial experiments, AI models, leveraging multi-omics data, can expedite the selection of suitable patients, leading to considerable time and cost savings. Additionally, these models can predict therapy outcomes, aiding in the decision-making process for patients contemplating the treatment, thereby optimizing resource utilization and enhancing overall therapy safety. Moreover, generative AI models can play a crucial role in synthesizing omics data, addressing challenges related to data quality and further contributing to cost-effectiveness in the gene editing landscape. Also, the development and maintenance of sophisticated AI models demand substantial financial resources. Additionally, the expenses related to laboratory equipment, reagents, and skilled personnel for CRISPR experiments contribute to the overall cost burden. Achieving a cost-effective balance between cutting-edge AI technologies and the practicalities of implementing CRISPR therapies remains a significant hurdle.

Another significant difficulty in CRISPR-Cas9 gene editing therapies is the development of effective delivery methods tailored to target specific tissues. Ensuring the precise and effective delivery of CRISPR components poses a significant technical challenge. There are three potential cargo forms: mRNA, DNA, and ribonucleoprotein combinations. These cargoes can be delivered through various methods such as viral carriers (e.g., lentivirus), liposomes, and physical methods like electroporation. AI has the ability to play a crucial role in optimizing cargo selection. It can aid in designing and refining delivery vehicles customized for specific tissues or cell types (Egorov et al., 2021). By analyzing patient data and genetic profiles, AI can assist in tailoring delivery methods to ensure precise and accurate targeting. However, challenges persist in achieving targeted delivery without off-target effects, requiring continuous refinement in both AI algorithms and experimental techniques.

The incorporation of AI models into healthcare practices underscores the vital importance of ethical considerations and compliance with regulations. It is imperative to prioritize patient privacy, maintain transparency in AI decision-making procedures, and adhere to ethical standards when deploying AI in gene editing within healthcare. This becomes particularly crucial when dealing with the accumulation of extensive patient data stored in the cloud, as safeguarding data protection and privacy emerges as a major concern. There is a need for robust regulations and ethical frameworks to prevent the exploitation of AI technologies for unauthorized or unethical gene editing practices. This includes addressing concerns related to designer babies, enhancement interventions, and other ethically sensitive applications. Considerations must be given to vulnerable populations, including those with limited decision-making capacity, such as minors or individuals with cognitive impairments. The ethical implications of using AI to guide gene editing in these cases involve ensuring informed consent, protecting autonomy, and avoiding undue influence in decision-making processes.

While there’s a growing body of AI research dedicated to BED (Jeong et al., 2020; Azameti and Dauda, 2021), and PED (Bhat et al., 2022; Capponi and Daniels, 2023), there’s a notable scarcity of AI models designed specifically for epi-GED. Furthermore, the existing AI models have not been trained to address CRISPR-based editing tools, including Cas12a and others (Ibrahim et al., 2022; Lee, 2023). AI holds great promise in unlocking the capabilities of these emerging gene-editing tools, potentially revolutionizing the field. CRISPR technology is a present-day reality with incredible potential. Establishing a comprehensive, centralized CRISPR database is imperative. This repository should encompass a wide range of data, covering different CRISPR tools, their applications across various use cases, and their relevance to different diseases. Such a database would serve as a catalyst for fostering collaboration between different disciplines in the field, accelerating progress and innovation. Another critical challenge is the safe introduction of *in-vivo* gene editing into clinical practice. AI is already proving valuable in predicting effective gRNAs and their potential off-target effects. These advancements will play a crucial part in ensuring the safe and responsible application of *in-vivo* gene editing in clinical settings in the future. Finally, ensuring the safety and reliability of AI models that can support real-time decision-making in clinical settings is also a challenge. Addressing the issues of clinical deployments, such as validation, security, and compliance with healthcare standards is important.

## 7 Research gaps and future research directions of AI’s application in genome editing

### 7.1 Optimizing Deep Learning Network Designs: Focus on Explainability and Interpretability

Developing effective DL network architectures and fine-tuning optimization hyperparameters is a critical but challenging task. AI models can be created that can help in automating the design and hyperparameter optimization for GED tasks.

As the importance of understanding DL networks grows, future research should continue to develop techniques for enhancing the explainability and interpretability of these models in the context of GED. This is crucial for therapeutic applications and understanding the mechanisms of on- and off-target activity. To gain insights into how each feature influences model predictions, future research should promote the use of interpretable model evaluation techniques like SHAP (Shapley Additive explanations) (Lundberg and Lee, 2017) and Tree SHAP (Lundberg et al., 2020). These algorithms can help in providing clear explanations for model behavior and credit allocation.

### 7.2 Transfer learning for short data sets

Most existing methods for developing predictive models in the CRISPR-Cas9 domain rely on a single dataset or a small number of gRNAs, leading to potential bias and insufficient predictive power. Future research should focus on strategies to address data sparsity issues by combining multiple datasets effectively and mitigating dataset-specific biases. One potential solution to the problem of insufficient training data is to utilize transfer learning. Future research should explore how to optimally select larger data sets for training DL models that can predict off-target sequences in short data sets using transfer learning. Additional research should demonstrate the criteria for choosing the best larger datasets for training. Coarse-grained high/low classifications are currently favored due to the small sample sizes and limited feature sets in CRISPR-Cas9 datasets. However, future research should aim to improve regression-based techniques to characterize gRNA efficiency more precisely, making it possible to predict the efficiency of gRNAs.

### 7.3 Utilizing informative features and uncertainty quantification

Greater accuracy in GED predictions can be achieved by adding informative factors such as RNA fold score, microhomology properties, and epigenetic features to the models. AI models can be developed to automate the identification of meaningful features from sequences, reducing potential biases that can be there during manual feature selection.

The role of uncertainty quantification in GED should be further explored. Researchers can investigate methods to assess both aleatoric and epistemic uncertainty, contributing to more accurate predictions for both on- and off-target regions in GED applications. In several study disciplines, this method has gained popularity for assessing uncertainty (Abdar et al., 2021a; Abdar et al., 2021b; Abdar et al., 2023; Hoffmann et al., 2021; Mazoure et al., 2022).

### 7.4 *In-silico* screens with improved models and expanding beyond on-target and off-target predictions

Ongoing work in ML approaches for protein structure modeling, including the integration of structural descriptors, can enhance the prediction of variations’ activities in CRISPR-Cas9 applications. Researchers should continue to improve *in silico* screening methods for more accurate predictions.

The off-target effects of CRISPR-Cas-based editing technologies must be minimized, and numerous AI models have been created to address this concern, providing a partial solution to the problem. Additionally, there is a requirement to predict the effects of different CRISPR-Cas9 implementations, such as knock-ins and base modifications, that go beyond the conventional on-target effects of gene knockouts or off-target prediction. Furthermore, AI structure prediction models have helped to create various versions of base and prime editors. Considering the modular nature of these editors, AI models can help in selecting the best combination for different applications.

## 8 Conclusion

GED technologies, particularly CRISPR-Cas9, have opened exciting possibilities for understanding genes and improving medical treatments. The integration of AI plays a vital role in enhancing the precision, efficiency, and affordability of GED, especially in addressing genetic diseases like Sickle cell anemia, characterized by severe vaso-occlusive crises or Thalassemia. AI models have been employed in designing gRNAs for CRISPR-Cas systems, widely used in GED technologies. Designing gRNAs is crucial for editing efficiency, and specificity, and avoiding off-target effects. AI models, including DeepCRISPR, CRISTA, and DeepHF, predict optimal gRNAs, considering factors like genomic context, Cas protein type, on-target/off-target scores, and the outcomes of GED. These models employ various ML and DL techniques, such as CNNs, Random forests, and SVMs, learning from extensive, high-quality datasets of gRNA sequences and their effects on GED. They provide valuable guidance for researchers conducting CRISPR-Cas genome editing experiments. AI-driven models also assist in designing and optimizing advanced GED techniques such as BED, PED, and epiGED. These techniques introduce precise, programmable changes to DNA sequences, eliminating the need for homology-directed repair pathways or donor DNA templates. AI models, like BE-Hive and PE-Design, select optimal editors for target sequences, accounting for genomic context, desired mutation types, off-target effects, and potential impacts on gene function and phenotype.

Furthermore, AI, in conjunction with Genome Editing and precision medicine, enables personalized treatments based on genetic profiles. It analyzes patients’ genomic data, identifying disease-associated mutations, variations, and biomarkers, such as those in cancer, diabetes, Alzheimer’s, and more. It predicts personalized treatment options, considering efficacy, toxicity, and resistance to various drugs and therapies. AI also monitors treatment response and adjusts it accordingly. AI models help design and optimize these GED-techniques, providing tools for predicting editing activity, specificity, efficiency, and outcomes. For instance, AI models assist in selecting optimal BED, PED, or epiGED for a given target sequence, considering genomic context, desired mutations, off-target effects, and potential impacts on gene function and phenotype. AI models also optimize the delivery and expression of GED components, such as Cas proteins, guide RNAs, reverse transcriptases, and epigenetic modifiers. They can help in designing efficient vectors, promoters, and enhancers, improving delivery specificity to various cell types and tissues. AI empowers PED by predicting pegRNA efficacy, insertion efficiency, and editing outcomes, enabling versatile and precise genetic modifications. AI-driven tools, like EpiCas-DL, predict sgRNA activity for epi-GED, regulating gene expression without altering DNA sequences. The convergence of AI, CRISPR, and precision medicine offers the potential for personalized treatments, effectively targeting individual genetic profiles. While AI has significantly advanced GED, challenges such as cost reduction, optimized delivery methods, safety in clinical deployment, and the need for comprehensive CRISPR databases remain to be addressed. Research in AI applications for GED should focus on areas such as transfer learning, network design optimization, explainability, informative feature selection, uncertainty quantification, and expanding beyond on-target/off-target predictions. AI’s pivotal role in GED presents innovative solutions to longstanding challenges, promising a future where gene editing is safer, more precise, and accessible for a broader range of medical applications. As technology continues to evolve, the synergy between AI and GED will continue to shape the field of genetics, biomedicine, and healthcare, with far-reaching implications for the betterment of human health.
