# Supplementary material for: Advancing genome editing with artificial intelligence: opportunities, challenges, and future directions
Source: Front Bioeng Biotechnol. 2024 Jan 8;11:1335901. doi: 10.3389/fbioe.2023.1335901 (PMC10800897; doi:10.3389/fbioe.2023.1335901)
Supplement: Supplementary file 1 [file DataSheet1.DOCX]

Supplementary Material

Advancing Genome Editing with Artificial Intelligence: Opportunities, Challenges, and Future Directions

**Shriniket Dixit ^1†^, Anant Kumar ^2†^, Kathiravan Srinivasan ^1^, P M Durai Raj Vincent ^3^, Nadesh R.K. ^3,^** *

^1^School of Computer Science and Engineering, Vellore Institute of Technology, Vellore 632014, India

^2^School of Bioscience and Technology, Vellore Institute of Technology, Vellore 632014, India

^3^School of Computer Science Engineering and Information Systems, Vellore Institute of Technology, Vellore 632014, India

† These authors have contributed equally to this work and share first authorship.

*** Correspondence:**Nadesh R.K.
rknadesh@vit.ac.in

## Supplementary Figure


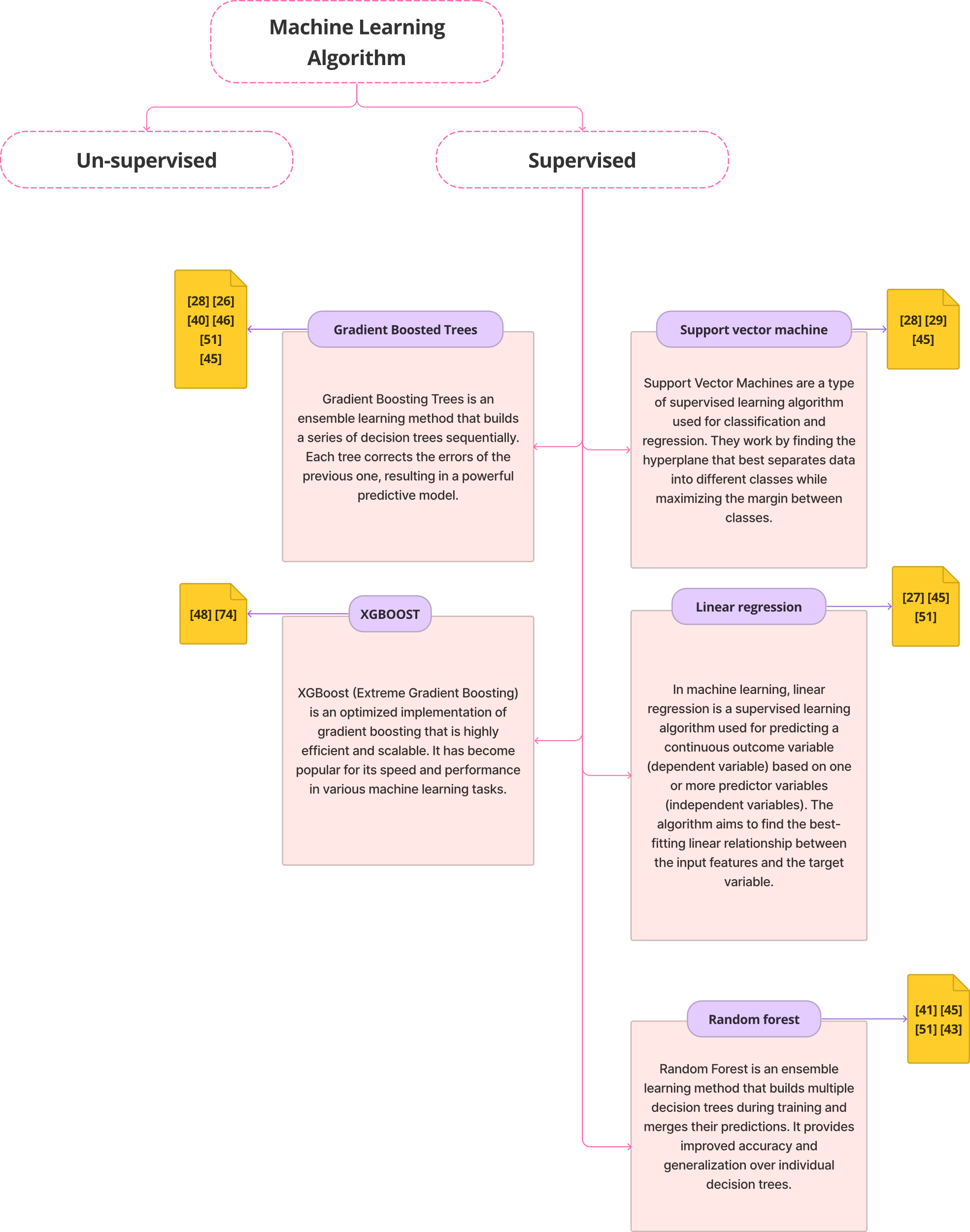


**Figure 1. ML algorithms used in this review**. ML algorithms, are typically categorized into two types - supervised and unsupervised. In this review, the focus is on tools (aid in genome editing) that predominantly use supervised ML algorithms, such as Support Vector Machines (SVM), Random Forests (RF), Gradient Boosted Trees (GBT), XGBoost, and linear regression. The blocks highlighted in yellow present a compilation of references wherein these tools have been applied and utilized. And the blocks in pink provides simple and brief explanation of these algorithms.


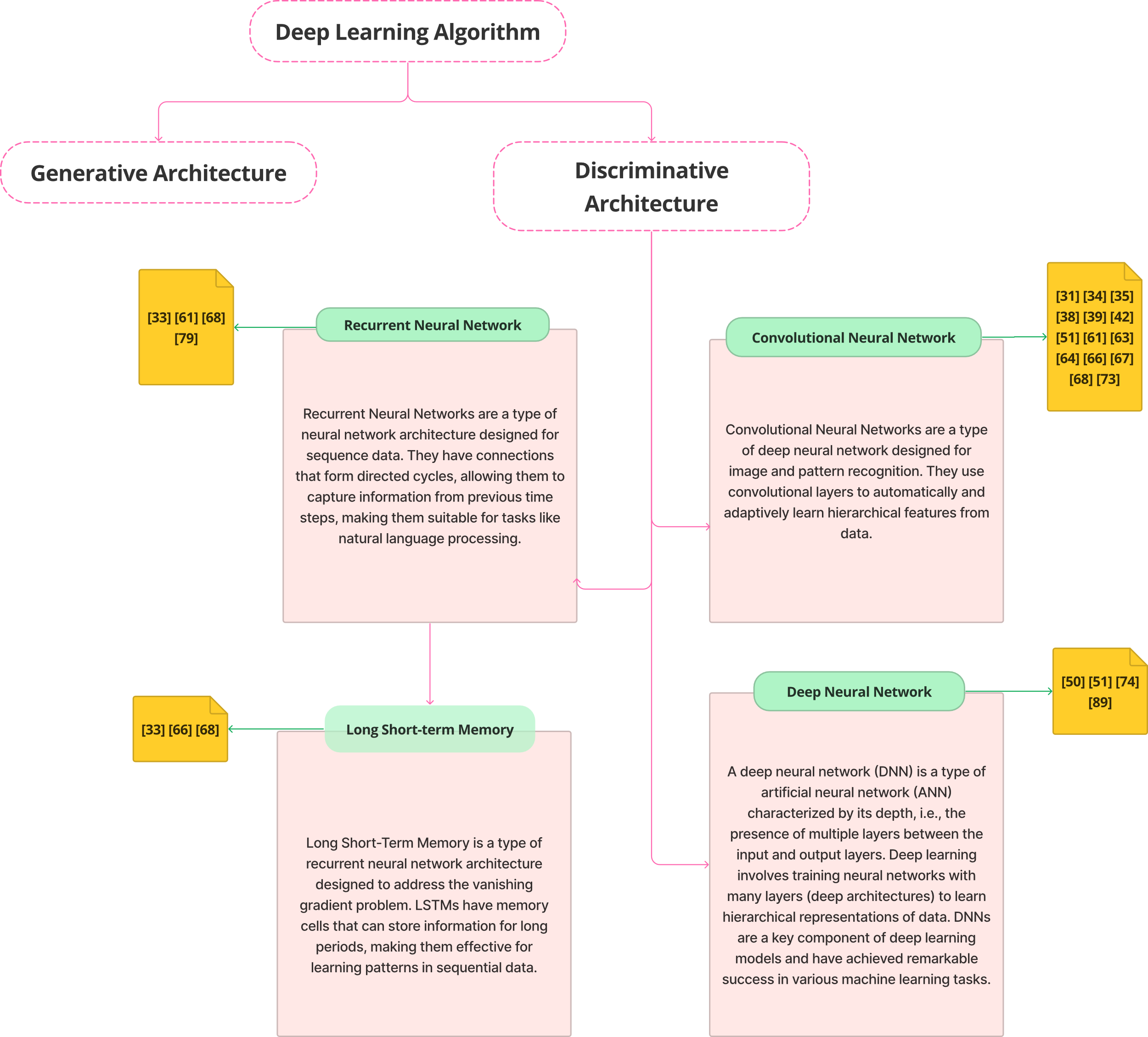


**Figure 2. DL algorithms used in this review**. DL algorithms, are typically uses two types of architecture- discriminative and generative. In this review, the focus is on tools (aid in genome editing) that predominantly use discriminative architecture DL algorithms, such as Convolutional Neural Network (CNN), Deep Neural Networks (DNN), and Recurrent Neural Network (RNN). The blocks highlighted in yellow present a compilation of references wherein these tools have been applied and utilized. And the blocks in pink provides simple and brief explanation of these algorithms.
